# Supplementary material for: Taxonomic and Gene Category Analyses of Subgingival Plaques from a Group of Japanese Individuals with and without Periodontitis
Source: Int J Mol Sci. 2021 May 18;22(10):5298. doi: 10.3390/ijms22105298 (PMC8157553; doi:10.3390/ijms22105298)
Supplement: Supplementary file 1 [file ijms-22-05298-s001.zip › Supplemental_FigureF_v2.pdf]

Supplementary material

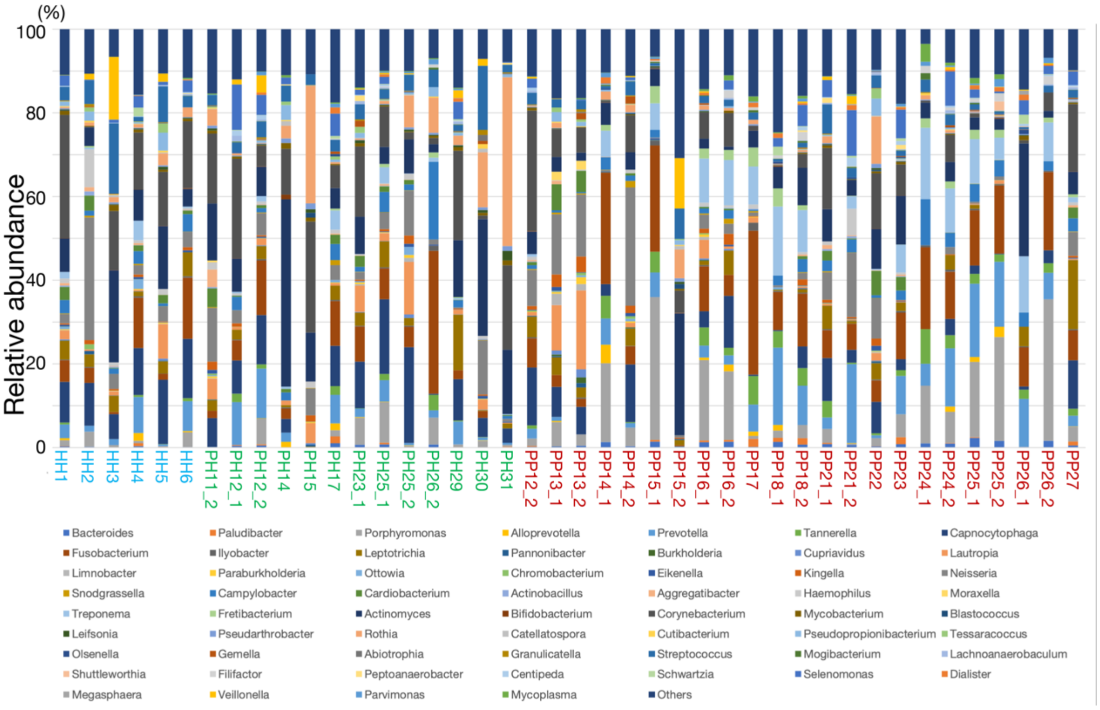

Figure S1. The genus-level taxonomic composition of all samples.

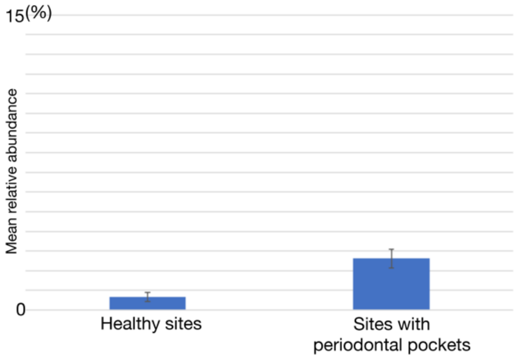

(**a**) *Tannerella*

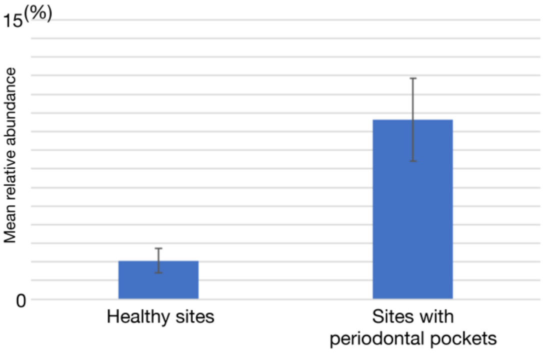

(**b**) *Porphyromonas*

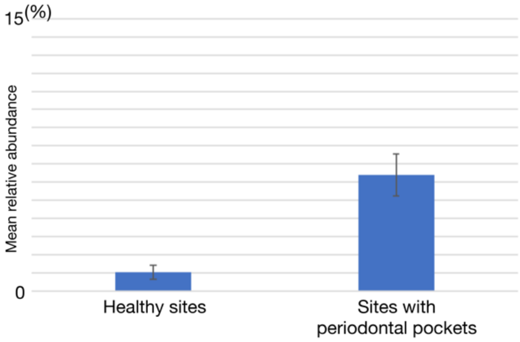

(**c**) *Treponema*

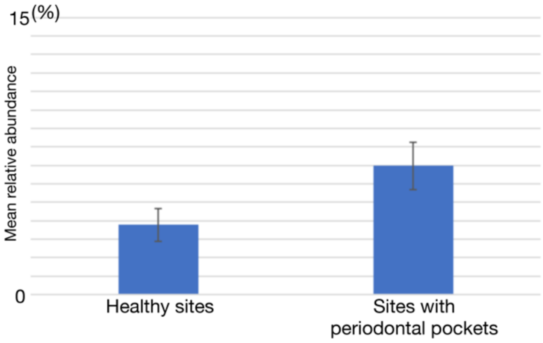

(**d**) *Prevotella*

Figure S2. The relative abundance of (**a**) *Tannerella*, (**b**) *Porphyromonas*, (**c**) *Treponema* and (**d**) *Prevotella*. Data are shown as the mean ± standard error of the mean.
