# Supplementary material for: Taxonomic and Gene Category Analyses of Subgingival Plaques from a Group of Japanese Individuals with and without Periodontitis
Source: Int J Mol Sci. 2021 May 18;22(10):5298. doi: 10.3390/ijms22105298 (PMC8157553; doi:10.3390/ijms22105298)
Supplement: Supplementary file 1 [file ijms-22-05298-s001.zip › Supplemental_Table_S2F-S5F_v3.pdf]

Table S2. Dominant genera at the healthy sites and sites with periodontal pocket

Healthy site	(%)	Site with periodontal pocket	(%)
Corynebacterium	13.01	*Fusobacterium*	11.52
Actinomyces	12.15	*Porphyromonas*	9.63
Capnocytophaga	9.28	*Prevotella*	6.97
Fusobacterium	6.77	*Actinomyces*	6.76
Rothia	6.16	*Treponema*	6.40
Neisseria	5.66	*Corynebacterium*	6.08
		Neisseria	5.05

Table S3. Genera showed high abundance at sites with periodontal pockets compared with healthy sites

Genera	Healthy site (%)	Site with periodontal pocket (%)
Bacteroides	0.28	0.86
Porphyromonas	2.29	9.63
Tannerella	0.65	2.62
Burkholderia	0.01	0.11
Moraxella	0.02	0.18
Treponema	1.01	6.4
Fretibacterium	0.16	1.32
Mogibacterium	0.07	0.2
Shuttleworthia	0	0.1
Filifactor	0.08	0.56
Megasphaera	0.02	0.06

Table S4. Genera showed high abundance at healthy sites in subjects with periodontitis compared with that in healthy subjects

Genera	Healthy subjects (%)	Subjects with periodontitis (%)
Abiotrophia	0.03	0.18
Dialister	0.05	0.27
Filifactor	0.01	0.11
Fretibacterium	0.07	0.21
Gemella	0.05	0.25
Mogibacterium	0.02	0.09
Moraxella	0.00	0.03
Olsenella	0.00	0.08
Parvimonas	0.04	0.32
Peptoanaerobacter	0.02	0.15
Rothia	1.38	8.37
